# Additional consensus recommendations for conducting complex innovative trials of oncology agents: a post-pandemic perspective

**DOI:** 10.1038/s41416-022-02051-7

**Published:** 2022-11-24

**Authors:** Sarah P. Blagden, Ly-Mee Yu, Stephanie Ellis, Helen Hughes, Abeer Shaaban, Jonathan Fennelly-Barnwell, Mark P. Lythgoe, Alison M. Cooper, Francois M. Maignen, Sean W. Buckland, Pamela R. Kearns, Louise C. Brown, Experimental Cancer Medicine Centres (ECMC) CID trials working group

**Affiliations:** 1 Department of Oncology, Old Road Campus Research Building, University of Oxford, Oxford, UK; 2 Primary Care Clinical Trials Unit, Nuffield Department of Primary Care Health Sciences, University of Oxford, Oxford, UK; 3 London Hampstead Research Ethics Committee, Health Research Authority, Elephant and Castle, London, UK; 4 Cardiff and Vale University Local Health Board, University Hospital of Wales, Cardiff, UK; 5 Queen Elizabeth Hospital Birmingham and the University of Birmingham, Birmingham, UK; 6 Imperial College London, Hammersmith Hospital, Du Cane Road, London, UK; 7 The Association of the British Pharmaceutical Industry, London, UK; 8 National Institute for Health and Care Excellence (NICE), London, UK; 9 Pfizer Ltd, Walton Oaks, Tadworth, Surrey, UK; 10 Cancer Research UK Clinical Trials Unit, NIHR Birmingham Biomedical Research Centre, Institute of Cancer and Genomic Sciences, College of Medical and Dental Sciences, University of Birmingham, Birmingham, UK; 11 MRC Clinical Trials Unit at University College London, 90 High Holborn, London, UK

**Keywords:** Drug development, Adaptive clinical trial

## Abstract

In our 2020 consensus paper, we devised ten recommendations for conducting Complex Innovative Design (CID) trials to evaluate cancer drugs. Within weeks of its publication, the UK was hit by the first wave of the SARS-CoV-2 pandemic. Large CID trials were prioritised to compare the efficacy of new and repurposed COVID-19 treatments and inform regulatory decisions. The unusual circumstances of the pandemic meant studies such as RECOVERY were opened almost immediately and recruited record numbers of participants. However, trial teams were required to make concessions and adaptations to these studies to ensure recruitment was rapid and broad. As these are relevant to cancer trials that enrol patients with similar risk factors, we have added three new recommendations to our original ten: employing pragmatism such as using focused information sheets and collection of only the most relevant data; minimising negative environmental impacts with paperless systems; and using direct-to-patient communication methods to improve uptake. These recommendations can be applied to all oncology CID trials to improve their inclusivity, uptake and efficiency. Above all, the success of CID studies during the COVID-19 pandemic underscores their efficacy as tools for rapid treatment evaluation.

Our paper “Effective delivery of Complex Innovative Design (CID) cancer trials—A consensus statement” contained guidance around the design and conduct of CID trials and was published in January 2020 just 2 weeks before the first case of coronavirus disease 2019 (COVID-19) was reported in the UK [1]. The subsequent pandemic has raised the profile of CID trials and had significant and lasting consequences for clinical research in the UK.

At the start of the pandemic, the EU Committee for Medicinal Products for Human use (CHMP) called for large-scale, international multi-arm clinical trials to determine the best treatments against SARS-CoV-2 upon which regulatory decisions could be made rather than relying on outcomes from multiple small studies or compassionate use programmes [2]. This led to the deployment of adaptive platform design trials to simultaneously assess multiple agents against a single control arm with flexible master protocols so new agents could be added as others completed assessment. Any concerns that they would be too cumbersome to address the therapeutic vulnerabilities of a rapidly mutating virus were dispelled when the largest of these, Oxford’s urgent public health (UPH) RECOVERY study, opened in April 2020 only nine days after its approval and, at the time of writing in 2022, has tested over a dozen novel and repurposed treatments in over 48,000 patients admitted to hospitals across the UK with proven or suspected SARS-CoV-2 infection [3]. Similarly, the PRINCIPLE trial which compares repurposed antiviral treatments administered in the community, took only fifteen days to open and has recruited over 11,700 people with active COVID-19 infection [4]. In addition, the successful CATALYST phase II platform trial highlighted the value of a biologically enriched CID study for testing the most effective therapeutic antibodies for further evaluation in late phase trials like RECOVERY [5].

Although these COVID-19 trials demonstrated the appropriateness of CID designs for delivering fast and efficient therapeutic research, their success was aided by four factors (1) the very large number of patients suddenly becoming available for research in an acute setting where no proven therapies existed, (2) the extensive and rapid redeployment of clinical research delivery staff and R&D office staff to conduct UPH studies, (3) the evaluation of predominantly repurposed drugs with relatively well understood safety profiles, and (4) the ability to use simple 28-day outcome measures. Although these factors are untypical of cancer trials, other innovative aspects of the large COVID-19 trials could be implemented into future cancer CID studies. For this reason, we have added three new recommendations to our ten original consensus statements (Table 1) which we will briefly describe here.

i. Employ pragmatism
  The RECOVERY trial team recognised that, in order for the study to be adopted by overstretched medical staff, any study-related activities must be minimal and (to quote the GCP guidance) “proportionate to the risks inherent in the trial and the importance of information collected” but without sacrificing scientific rigour [6]. GCP training was required only for those acting outside their usual responsibilities, all study materials were made available online and a 24-hour telephone service, manned by medical and trial coordinating centre staff, provided immediate solutions to queries. Baseline eligibility was confined to a single sheet, patient information sheets contained only focused information, consent was collected electronically and patient outcome information (such as mortality or intensive care hospitalisations etc.) was extracted from multiple clinical datasets obtained via NHS DigiTrials. There was a purposefully simple primary study endpoint of 28-day mortality and patients were randomised to one of four treatment arms or “no additional treatment” control.
  Study outcomes were transparent and publicly released in real-time as trial arms closed. For example, hydroxychloroquine failed to improve on the 25–30% and 30–40% mortality rate observed in hospitalised and ventilated patients respectively, whereas 10 days of dexamethasone was quickly shown to be beneficial [7, 8]. The subsequent rapid approval of dexamethasone as a treatment for severe COVID-19, in a time of disinformation and dubious COVID “cures”, was particularly remarkable and is estimated to have saved 1 million lives within the subsequent nine months. The second wave of the pandemic saw the RECOVERY study gain complexity and adopt an adaptive recruitment scheme but the original study endpoints were maintained.
  While it is unrealistic to assume that cancer studies will replicate these rates of approval and uptake, or that simple endpoints are possible (secondary and exploratory endpoints are often required in cancer studies for registration decisions), the RECOVERY trial highlights the importance of staying focused on a central research question, collecting only the minimum required dataset, providing study materials online, remote monitoring of patients where possible, using streamlined processes to reduce bureaucracy and improve engagement amongst staff, and releasing results quickly to change practice.
ii. Minimise negative environmental impacts
  Another focus is the environmental impact of clinical studies [9]. Out of necessity during the pandemic, many clinical trials teams held meetings virtually. The surprising success of this approach and the positive environmental impact of reducing the travel and carbon footprint of face-to-face meetings, for example in developing the SPIRIT Path extension recommendations, should be adopted as standard practice wherever possible [10].
  Large platform studies, although efficient in replacing multiple smaller trials, can generate huge carbon footprints from accumulating extensive paperwork and databases. We recommend the use of paperless systems with online trial master files and documents and databases linked to secure cloud repositories. For example, the electronic data capture system used in PANORAMIC, a community-based platform trial to evaluate antiviral treatments in COVID-19, was configured to allow participants to directly enrol onto it. This enabled the study team to recruit over 25,000 participants within 5 months.
iii. Use direct-to-patient approaches to improve enrolment

**Table 1:** A summary of the ten original and three new consensus recommendations.

Consensus recommendations	Summary
1. Trial Planning and Design: Engagement with Regulators	Joint meetings with regulators, HTA bodies and other key stakeholders to develop clinical trial protocol and shape delivery
2. Protocol Development	Seek to include all possible future modifications (e.g., additional study arms) to reduce need for any substantial amendments.
3. Patient and Public Involvement	Early and continued engagement during the planning, conduct and reporting
4. Patient Facing Documentation	Provide all patients with an invitation document, study specific document and an overview handbook
5. Statistical Considerations	Statistical input with flexibility to incorporate individual variation for differing treatments, diseases and molecular characterisations
6. Leadership and Oversight	Trial management group with experience of CID trials should be utilised
7. Dissemination of Results	Ensure the timely reporting of results
8. Staff Training	Ensure all staff trained in complex trial methodologies to they are confident in the delivery and conduct
9. Approval and Reimbursement Decisions	Accelerated Access initiatives are essential to ensuring CID trials are rapidly approved by regulators and reimbursed by HTA bodies
10. Evaluating the impact on Public Health	Consideration for wider future analysis of the impact of CID trials on public health
**Additional Post-pandemic Recommendations**	
11. Employ pragmatism	Proportionate staff training, self enrolment and electronic consent (where appropriate), focused endpoints and transparent outcomes
12. Minimise negative environmental impacts	Virtual meetings, paperless systems and secure, cloud-based databases
13. Direct-to-patient approaches	Explore methods for enhancing enrolment, work with communities to address uptake in underserved communities, involve relevant PPI groups to review patient-facing information

The pandemic highlighted health and social inequalities and prompted a renewed impetus towards improving inclusivity in clinical trials. To identify potential participants for the PRINCIPLE trial, many of whom were not linked to a GP and were home-isolating with active infection, the team worked with NHS 111 and NHS Digital, as well as publicising the study through national media outlets to encourage self-enrolment. Study drugs were couriered directly to patients’ homes from the central clinical trials unit. This direct-to-patient approach could be applied to future cancer CID studies where patient travel may be complicated by economic, practical (e.g. work or caring responsibilities) or disease-related reasons (e.g. fatigue or other symptoms due to underlying cancer).

To improve acceptance amongst participants from diverse ethnic backgrounds, or those living in areas of highest deprivation who tend to be underserved in clinical trials, the PRINCIPLE team also worked closely with local pharmacies, community organisations and religious leaders [11]. As there is considerable overlap in the conditions that predispose to cancer and severe COVID-19, such as economic deprivation, obesity, diabetes and chronic immunosuppression, the methods adopted by the PRINCIPLE investigators are highly relevant to future cancer CID trials [12]. Even with these measures in place, the azithromycin arm of the PRINCIPLE study had low numbers of Black participants (seven or 0.5% of the 2265 enrolled) suggesting more could be done to improve acceptance [13].

In our original consensus paper, we highlighted the importance of patient and public involvement (PPI) in the design and conduct of CID studies. It is vital that PPI is itself representative of underserved communities and that recruitment plans, protocols and patient-facing documents are scrutinised for factors such as eligibility criteria that might unwittingly exclude certain groups and widen cancer inequalities further still. The UK has national organisations offering PPI support, such as the NCRI Consumer Forum, ECMC PPI Group, and the NIHR Patient Engagement in Clinical Development Service for commercial sponsors. Representative PPI input can also ensure patient information for CID studies is comprehensible, ideally including figures such as route maps or links to videos that clearly explain the study design and treatment arms planned.

## Conclusions

Despite the horror and tragedy of the COVID-19 pandemic, the clinical research community has gained important insights into how CID studies can be run rapidly and at scale. As centres recover from the impact of the pandemic, and CID studies are once again launched to address the most prescient questions in cancer research, we have added three consensus recommendations to our original ten: employing pragmatic methods for CID trial conduct; minimising negative environmental impacts and using direct-to-patient approaches to improve enrolment. However, cancer trials are still beset by restraints to NHS workforce, research resourcing and prioritisation that need to be addressed to fully realise the potential of CID trials. Notwithstanding these limitations, insights from the major pandemic trials can be applied to conducting future CID studies and, ultimately, delivering new medicines to our cancer patients.

## Data Availability

Not applicable.
